# Supplementary material for: Platinum-nickel alloy excavated nano-multipods with hexagonal close-packed structure and superior activity towards hydrogen evolution reaction
Source: Nat Commun. 2017 Apr 24;8:15131. doi: 10.1038/ncomms15131 (PMC5413976; doi:10.1038/ncomms15131)
Supplement: Supplementary Information — Supplementary figures, supplementary tables, supplementary notes and supplementary references. [file ncomms15131-s1.pdf]

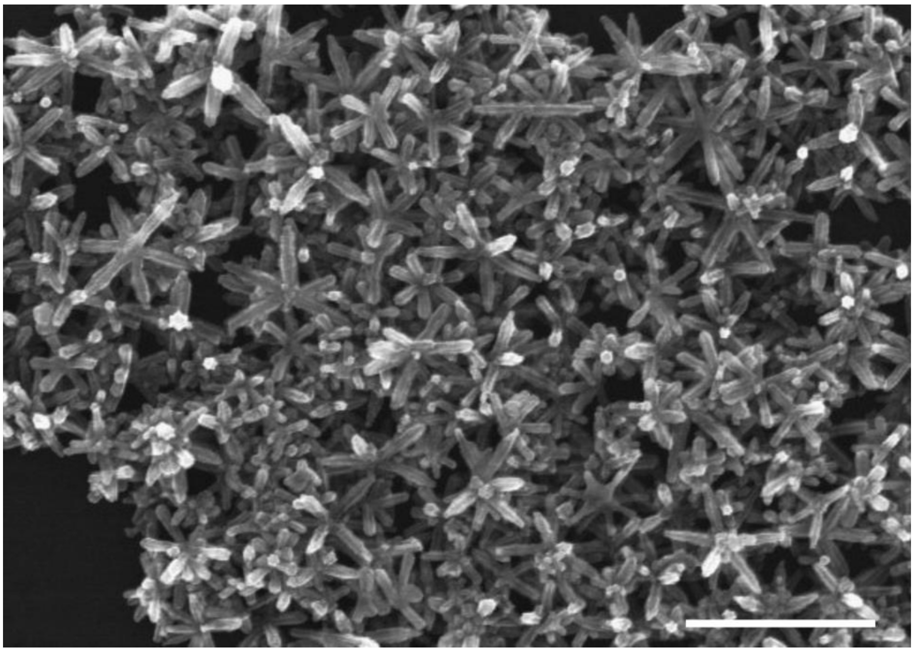

Supplementary Figure 1. SEM image of the Pt-Ni excavated nano-multipods (scale bar: 500 nm).

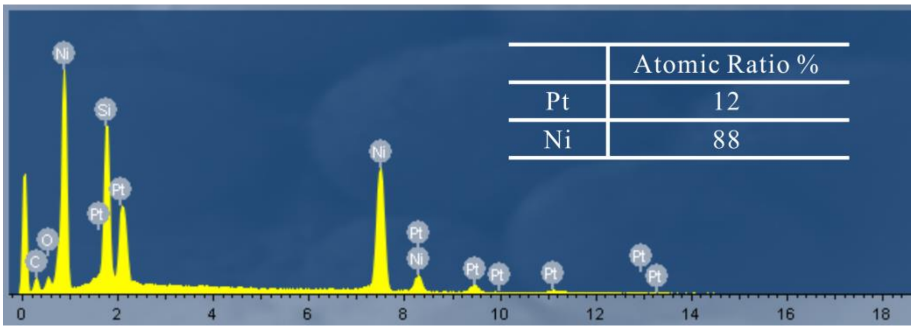

Supplementary Figure 2. Energy-dispersive X-ray spectroscopy of the Pt-Ni excavated nano-multipods as shown in Supplementary Figure 1.

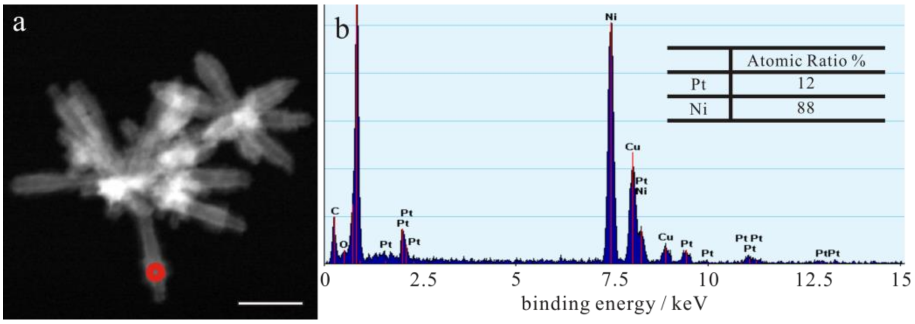

Supplementary Figure 3. (a) HAADF-STEM image of the Pt-Ni excavated nano-multipods at low magnification. (b) EDS of the selected area on the single branch in (a). Supplementary Figure 2-3 indicate that the Pt and Ni atoms are uniformly distributed throughout the whole nano-multipods.

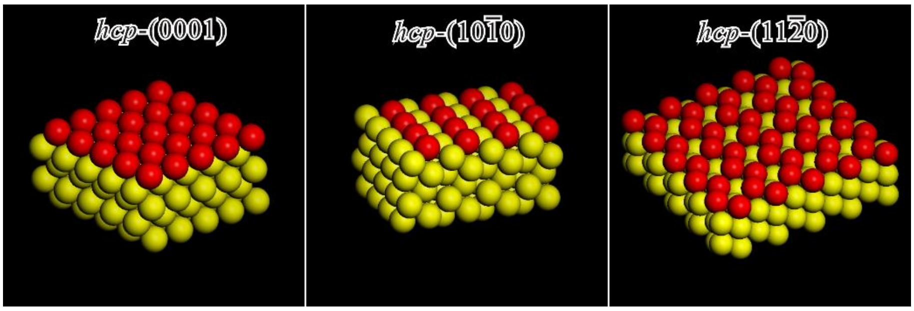

Supplementary Figure 4. Atomic arrangement of different crystal facets for the *hcp* crystal structure. The red balls represent the top layer atoms. For the *hcp* crystal structure, the coordination number of metal atoms in (0001), (10$\bar{1}$0) and (11$\bar{2}$0) facets are 9, 8 and 7, respectively. Therefore, the (11$\bar{2}$0) crystal facets in an *hcp* structure are of the highest surface energy among the three basic crystal facets (i.e. (0001), (10$\bar{1}$0) and (11$\bar{2}$0) facets).

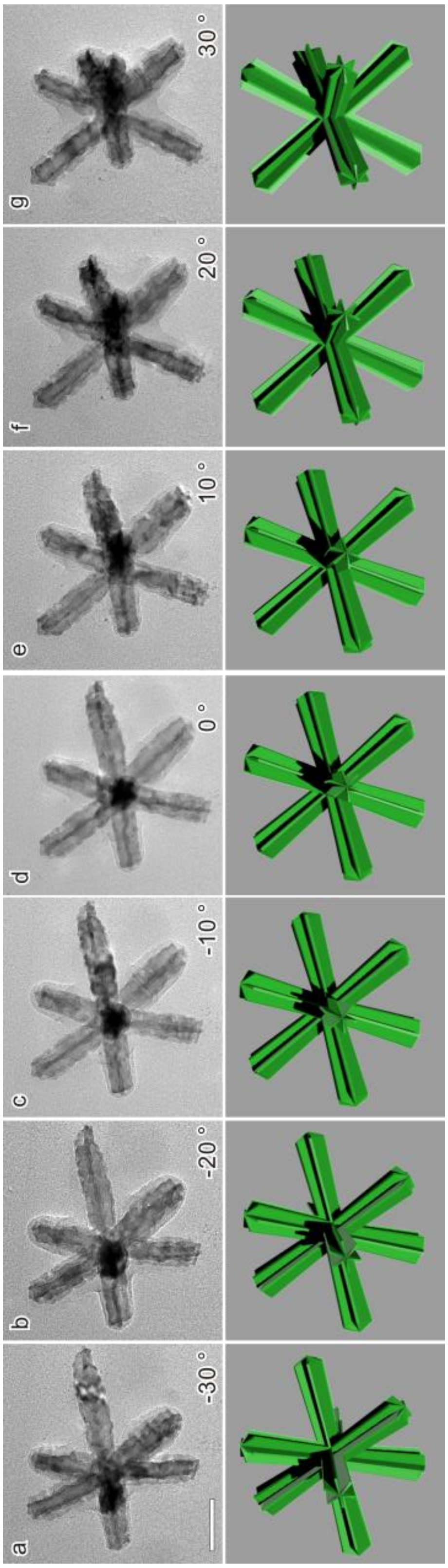

Supplementary Figure 5. TEM images and the corresponding schematic models of a single Pt-Ni excavated nano-multipod viewed from different orientations, (a-g) correspond to different tilt angles of TEM sample holder (from -30 ° to 30 °). Scale bar is 50 nm.

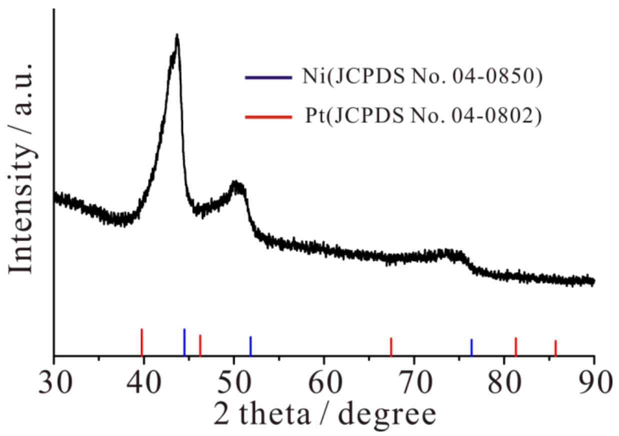

Supplementary Figure 6. XRD pattern of the product obtained from heat-treatment of the as-prepared *hcp* Pt-Ni nano-multipods at 350 °C under atmosphere of 5% H_2 and 95% N_2. The XRD pattern can be indexed as an *fcc* Pt-Ni alloy phase. The red and blue rod lines represent the diffractions of standard *fcc* Pt and *fcc* Ni phases, respectively.

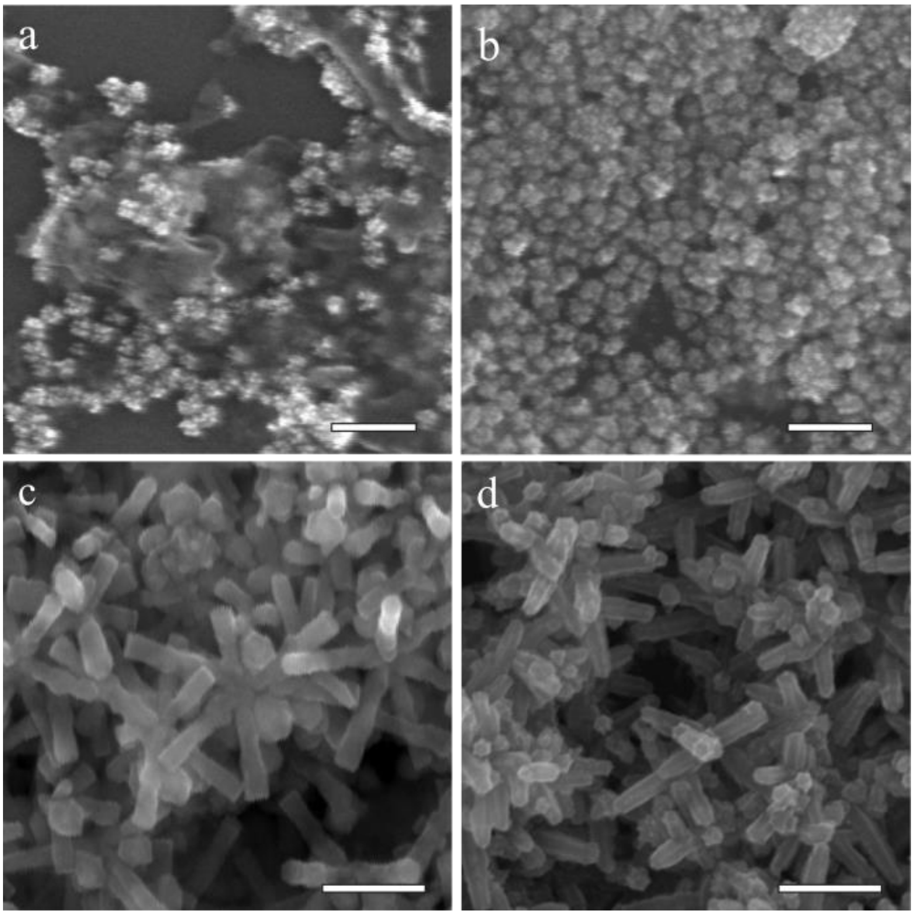

Supplementary Figure 7. (a-c) SEM images of the Pt-Ni products synthesized by adding different amounts of formaldehyde solution (with the reaction time of 12 h), (a) 0 μL, (b) 200 μL and (c) 400 μL. (d) SEM image of the Pt-Ni products synthesized from 400 μL formaldehyde solution with the reaction time of 36 h. Scale bars are 100 nm. It can be seen that the solid nano-multipods formed when 400 μL of formaldehyde solution was added and the reaction time was 12 h. However, the solid nano-multipods may also change to excavated ones when the reaction time prolonged from 12 h to 36 h. Therefore, the Pt-Ni excavated nano-multipods were also the final products when 400 μL formaldehyde solution was added.

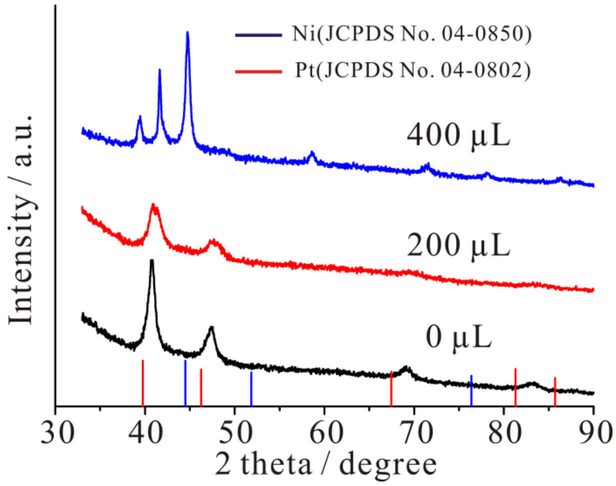

Supplementary Figure 8. XRD patterns of Pt-Ni NCs synthesized from different amounts of formaldehyde solution with reaction time of 12 h. The red and blue bars on the 2-theta axis represent the diffractions from standard *fcc* Pt and *fcc* Ni phases, respectively.

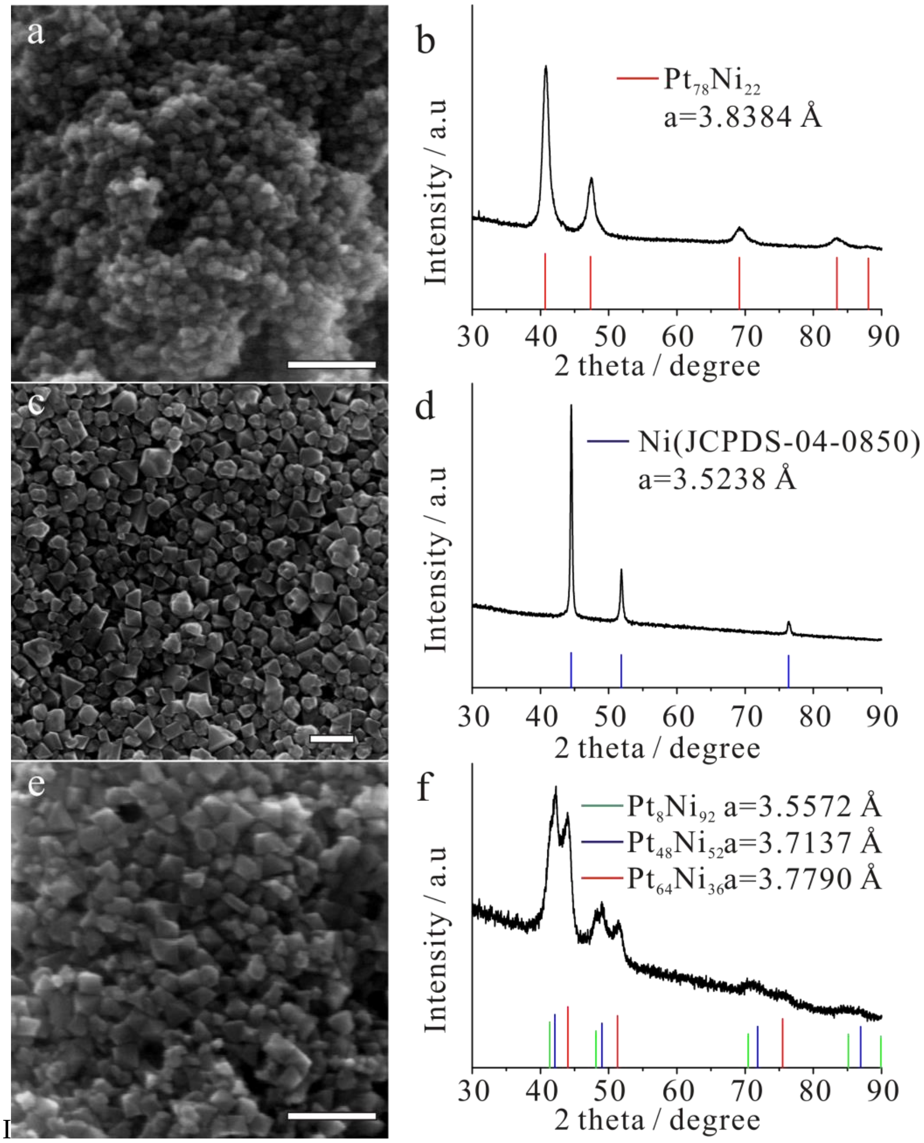

Supplementary Figure 9. SEM images and XRD patterns of the Pt-Ni products synthesized by replacing formaldehyde with (a and b) CO at 0.35 Mpa, (c and d) H_2 at 0.35 Mpa, and (e and f) mixture of CO and H_2 at 0.35 Mpa. Scale bars are 100 nm, 200 nm and 100nm for a, b and c, respectively. The vertical bars on the 2-theta axis of XRD pattern represent the calculated diffraction peaks of the corresponding *fcc* Pt-Ni products, respectively.

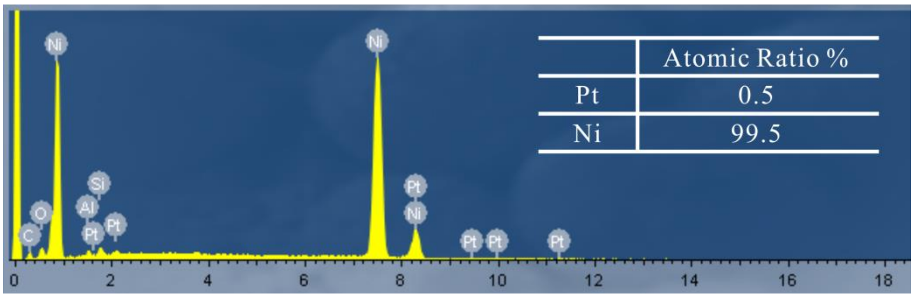

Supplementary Figure 10. The energy-dispersive X-ray spectroscopy of the product prepared from H_2 at 0.35 Mpa.

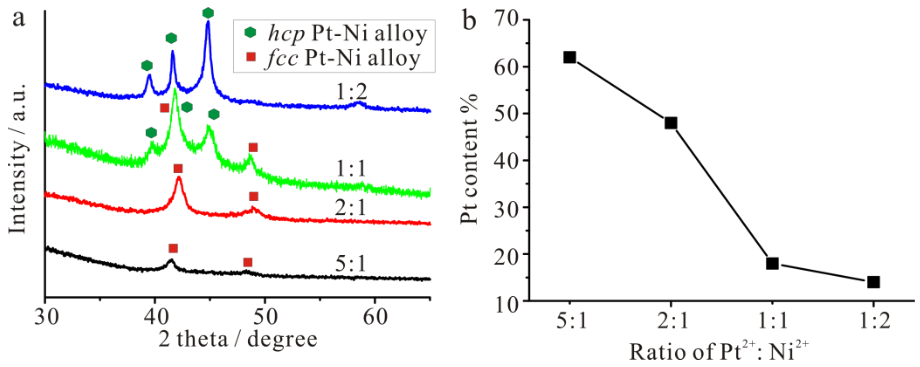

Supplementary Figure 11. (a) XRD patterns and (b) the corresponding Pt content in the final products which acquired by changing the molar ratio of $Pt(acac)_2$ and $Ni(acac)_2$ (Other reaction conditions are kept the same as typical reaction conditions for excavated *hcp* Pt-Ni nano-multipods).

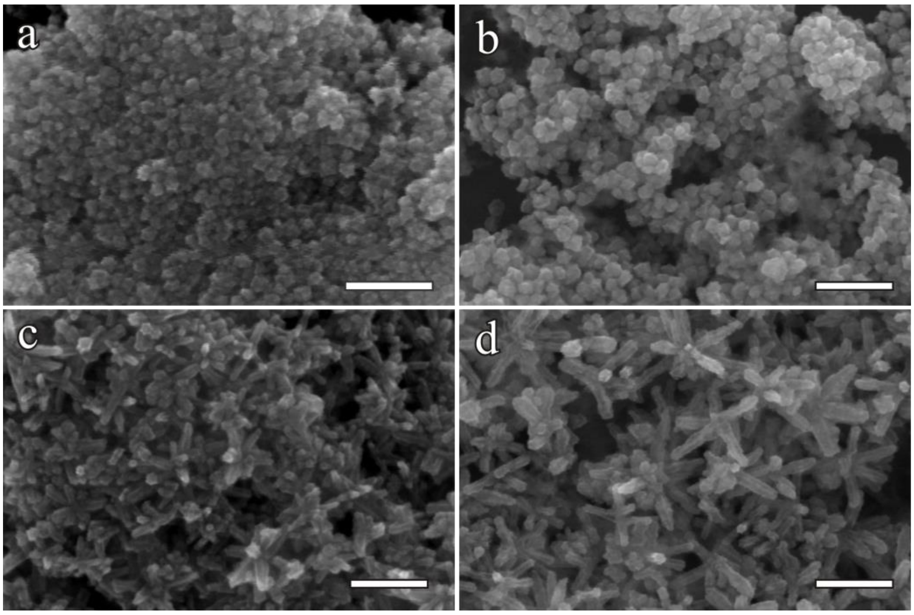

Supplementary Figure 12. SEM images of the corresponding products formed from different molar ratio between Pt(acac)$_2$ and Ni(acac)$_2$. (a) 5:1, (b) 2:1, (c) 1:1, (d) 1:2. Scale bars are respectively 150 nm, 200 nm, 200 nm and 200 nm.

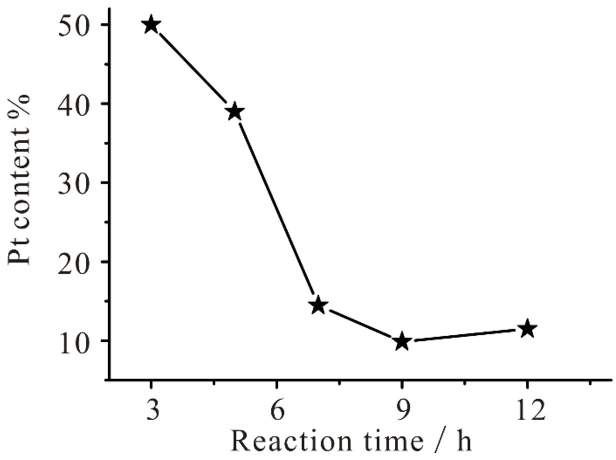

Supplementary Figure 13. The Pt contents of the as-prepared Pt-Ni NCs obtained from different reaction times.

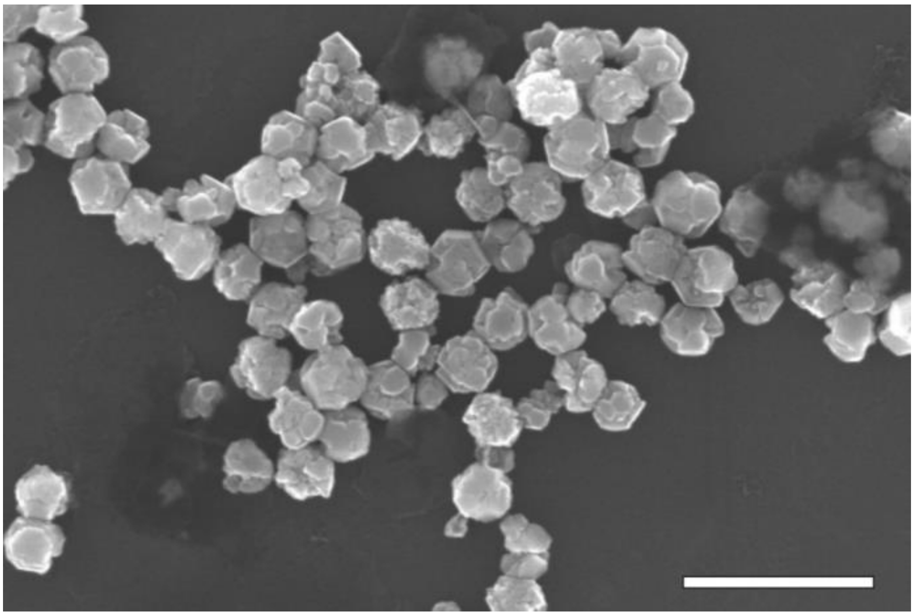

Supplementary Figure 14. SEM image of the product obtained in the absence of oleic acid while keeping other experimental conditions unchanged. Scale bar: 500 nm.

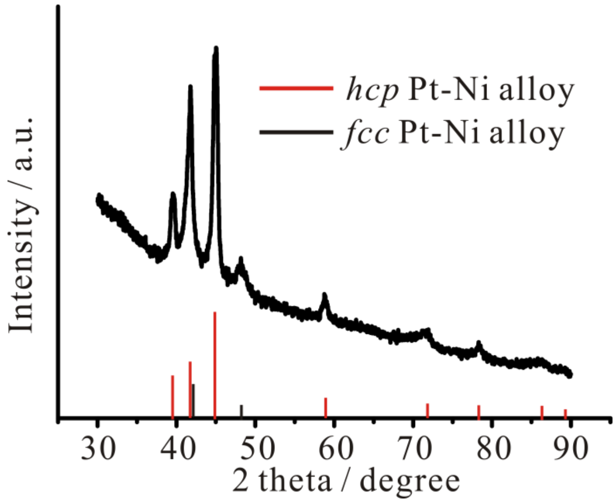

Supplementary Figure 15. XRD pattern of the product obtained in the absence of oleic acid while keeping other experimental conditions unchanged. The red and black rod lines represent the calculated diffraction peaks of the corresponding *hcp* and *fcc* Pt-Ni products, respectively. It can be seen that the branches cannot be formed in the absence of oleic acid. The oleic acid may change the crystal growth kinetics by selectively absorbing on the side face of the branch along the c-axis of *hcp* crystal structure, resulting in the formation of the multipods.

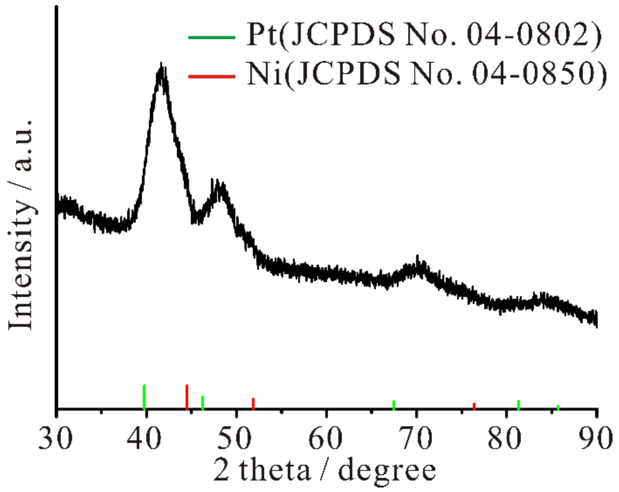

Supplementary Figure 16. XRD patterns of the Pt-Ni products synthesized by replacing formaldehyde with H_2 at 0.35 Mpa and oleylamine with octdecene. According to *Vegard*'s law, the alloy phase was determined to be $Pt_{62}Ni_{38}$ (Pt-rich). Combining with the results of oleylamine serving as solvent (Supplementary Figure 9c and 9d), it can be found that Pt atoms could keep preferential reduction and deposition in a solvent with weak coordination ability.

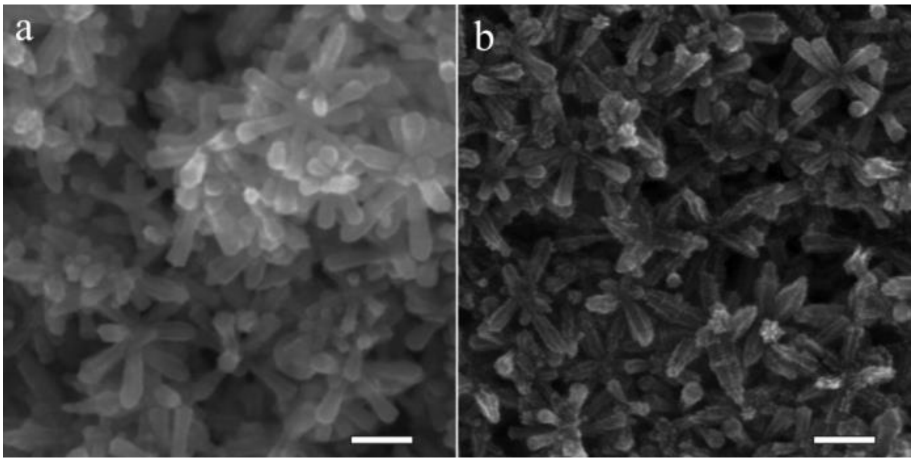

Supplementary Figure 17. SEM images of (a) the solid Pt-Ni alloy nano-multipods obtained from 9 h of reaction and (b) the Pt-Ni excavated nano-multipods obtained from 3 h more reaction time in the presence of $Pt(acac)_2$. Scale bars are 150 nm.

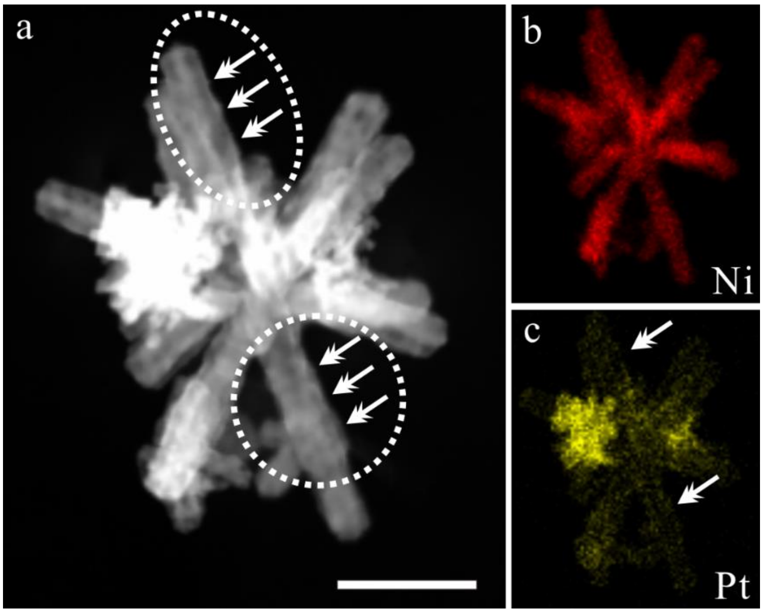

Supplementary Figure 18. HAADF-STEM and element mapping images of the solid Pt-Ni alloy nano-multipods obtained from 9 h of reaction. Scale bar is 100 nm. We can see that the contrast of the edge of single branch is slightly brighter than other parts. In addition, the elemental mapping of Pt element also shows the edge areas are rich of Pt atoms.

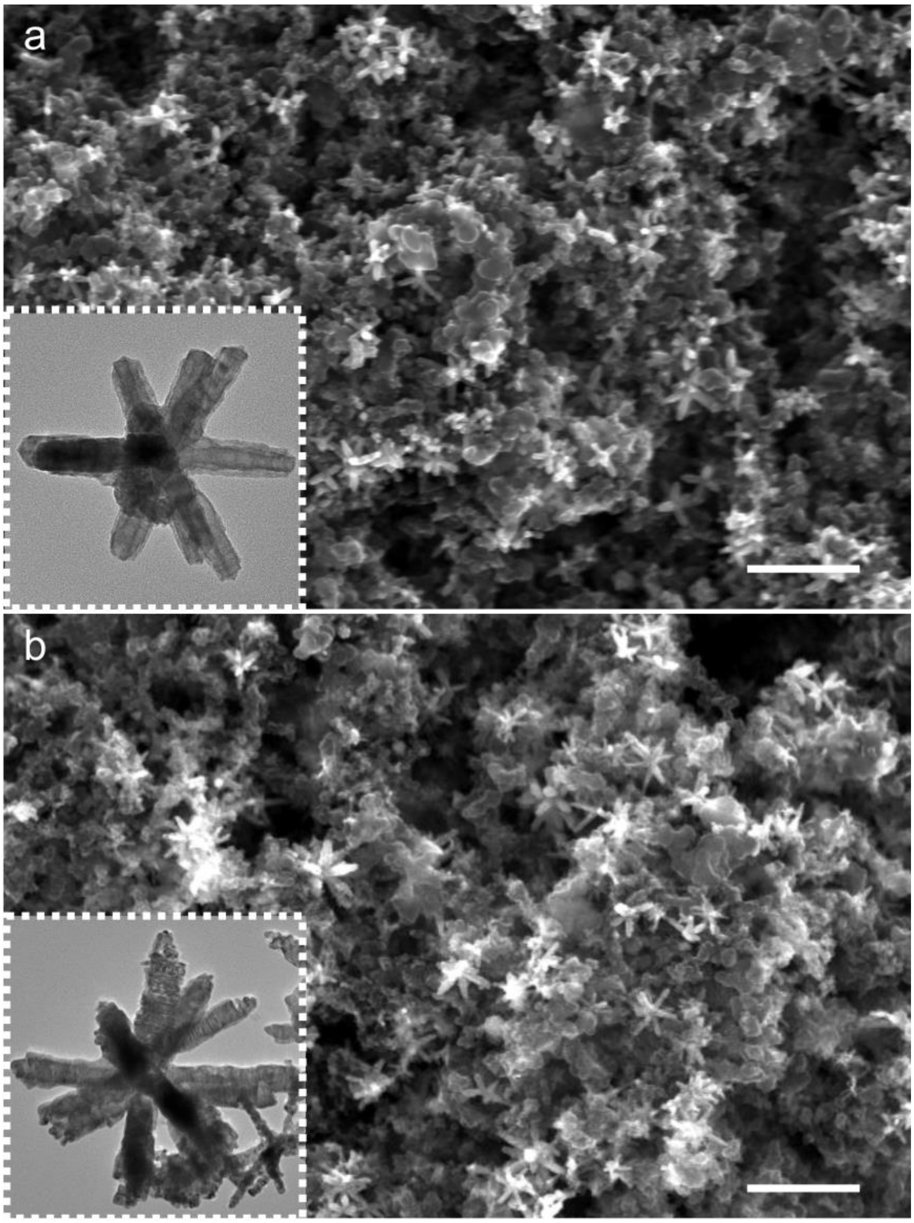

Supplementary Figure 19. (a) SEM image of the *hcp* Pt-Ni excavated nano-multipods supported on XC-72. (b) SEM image of the *fcc* Pt-Ni excavated nano-multipods supported on XC-72 obtained by heat-treating the *hcp* sample at 350 °C in the atmosphere of 5% H_2 and 95% N_2. Insets are TEM images of single excavated nano-multipod of the corresponding samples. Scale bars are 500 nm.

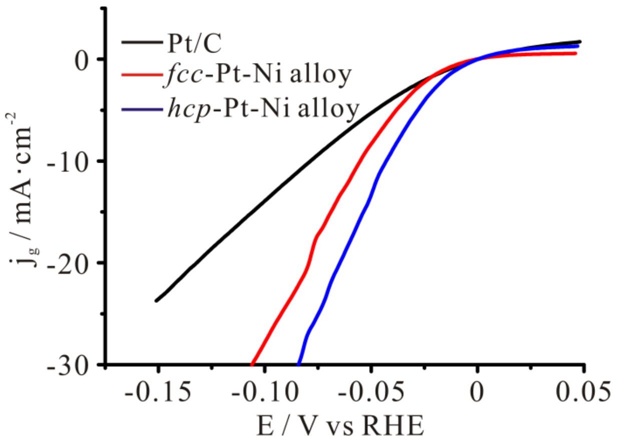

Supplementary Figure 20. The HER polarization curves of three catalysts in 0.1 M KOH normalized by the geometric area of the electrode.

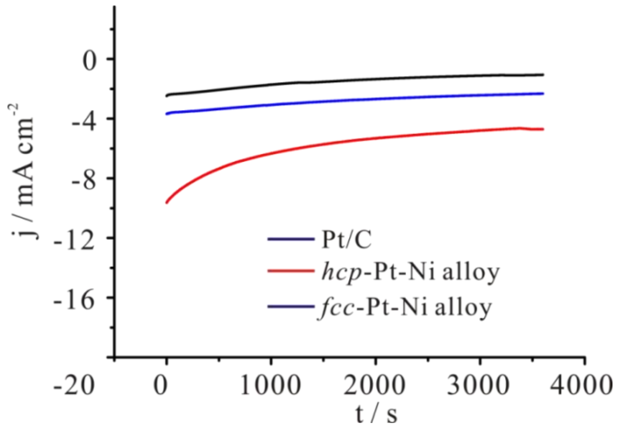

Supplementary Figure 21. *i-t* curves of three catalysts in 0.1 M KOH at potential of 65 m V *vs* RHE.

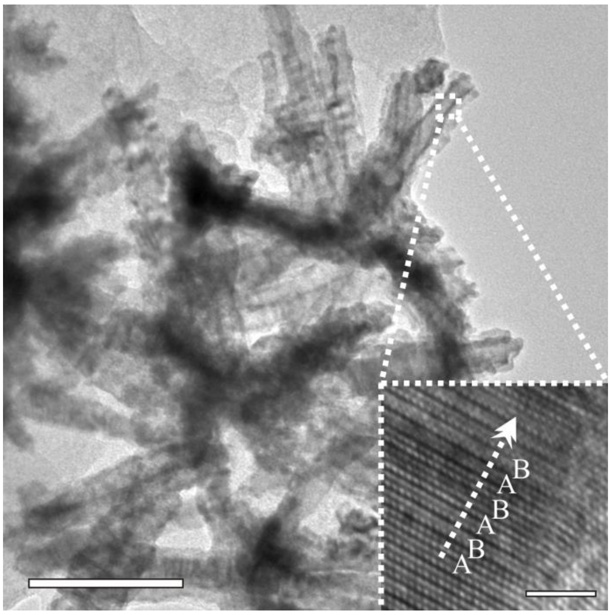

Supplementary Figure 22. TEM and HRTEM images (the inset) of the *hcp* Pt-Ni excavated nano-multipods after the HER test. Scale bars are 100 nm and 2 nm for TEM and HRTEM images respectively. It can be found that the *hcp* Pt-Ni excavated nano-multipods still remain the ABABAB packing mode after HER test, indicating that the *hcp* structure remained unchanged.

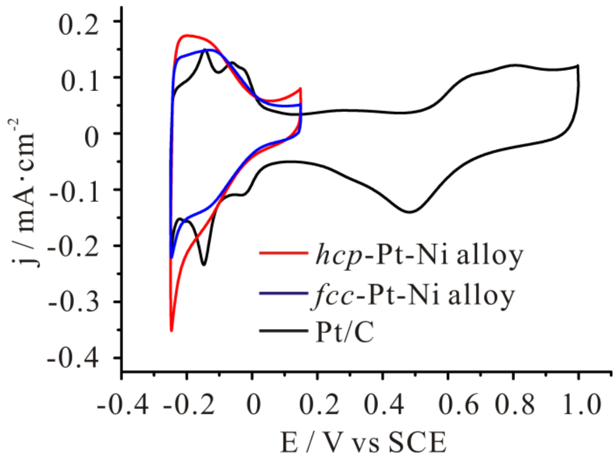

Supplementary Figure 23. CV curves of the catalysts in 0.1 M $HClO_4$. Scanning rate is 100 mV • s^{-1}. It should be noted that the upper limit potentials for Pt-Ni alloy in 0.1M $HClO_4$ was set to 0.15 V *vs* SCE (which was not as high as that of Pt/C), in order to prevent Ni in the Pt-Ni alloy from dissolution in the acid solution.

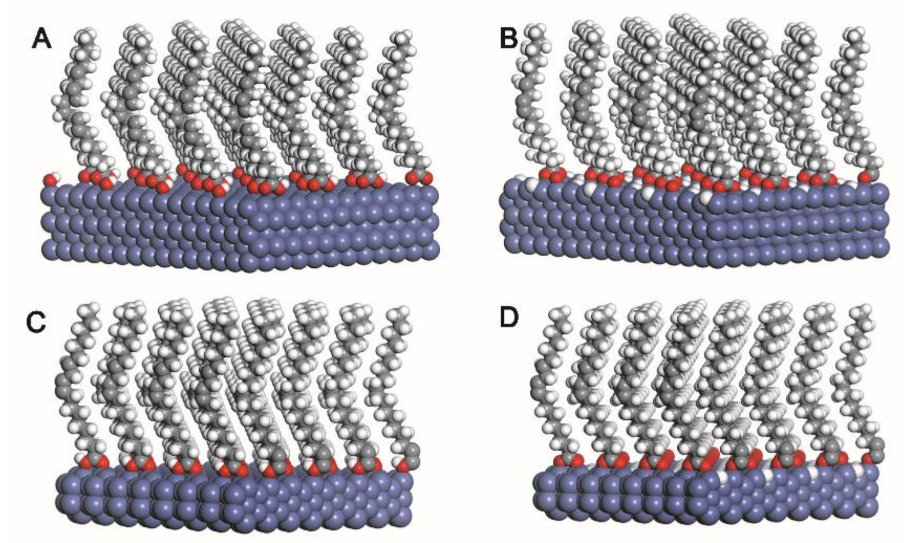

Supplementary Figure 24. Optimized structures of the adsorptions of OAH: (A) (B) molecular and dissociated adsorption on Ni {0001}; (C) (D) molecular and dissociated adsorption on Ni $\{11\bar{2}0\}$.

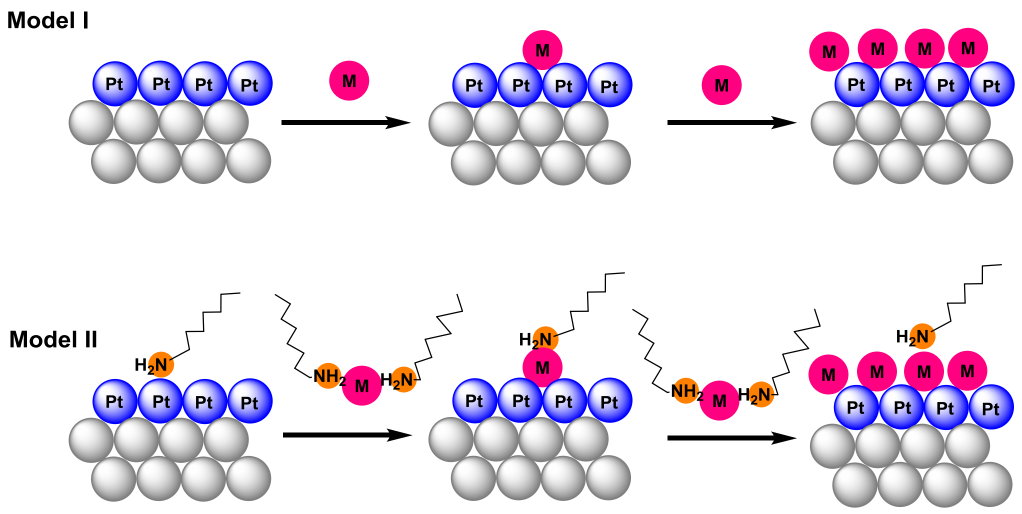

Supplementary Figure 25. Possible models for Pt or Ni deposition on the Pt skin surface.

Supplementary Table 1. Refined structure parameters for the *hcp* Pt-Ni alloy.

Crystal phase	Weight percent %	Crystal structure	Lattice parameters (Å)	Statistical parameters
$Pt_{12}Ni_{88}$	99.8	$P6_3/mmc$	a=2.6367<br>c=4.3310	R_p=2.05<br>R_{wp}=2.97<br>GOF=2.80
$Pt_{56}Ni_{44}$	0.2	$Fm\bar{3}m$	a=3.7484	

Supplementary Table 2. Calculated adsorption energies of oleic acid (OAH) on the Ni{0001} and Ni{$11\bar{2}0$} surfaces (Unit: eV/OAH).

Surface	Ni{0001}	Ni{$11\bar{2}0$}
OAH	-0.14	-0.39
OA+H	-1.45	-1.71

Supplementary Table 3. Calculated deposited energies of Pt or Ni atoms on PtNi alloy with Pt skin. (Unit: eV/atom)

Possible models	Ni		Pt	
	1/9 ML	1.0 ML	1/9 ML	1.0 ML
Model I	-3.65	-4.49	-3.82	-5.35
Model II	-1.40	-1.45	-0.59	-1.30

.

Supplementary Table 4. Summary of the typical catalytic performances of different catalysts

sample	*m*	j_o	j_s	j_g	j_m	η	References
Pt islands/Pt(111) surface	N/A	N/A	1.4	N/A	N/A	~140	1
$Ni(OH)_2$/Pt islands/Pt(111) surface	N/A	N/A	~2.2	N/A	N/A	~110	1
Pt (pc) electrode	N/A	0.7	2.4	N/A	N/A	N/A	2
$Ni(OH)_2$/modified Ir surface	N/A	N/A	~5.4	N/A	N/A	~61	3
$Ni(OH)_2$/modified Pt surface	N/A	N/A	~2.7	N/A	N/A	~95	3
Pt electrode	N/A	0.62	N/A	~5.0	N/A	~70	4
Pt_3Ni frames/C	N/A	N/A	2.3	N/A	N/A	84	5
Pt_3Ni frames/$Ni(OH)_2$/C	N/A	N/A	~6.0	N/A	N/A	~59	5
N, P doped-graphene	204	3.96*10^{-10}	~0	0	N/A	~570	6
Graphitic-C_3N_4/N-doped graphene	100	3.5*10^{-7}	N/A	~0	N/A	~560	7
Pt nanowires/SL-$Ni(OH)_2$	16 μg_{Pt} cm^{-2}	N/A	6.31	25.6	1.59	57.8	8
Ni-Ag alloy	N/A	N/A	~0	N/A	N/A	~260	9
Pt/C/20 wt% SL $Ni(OH)_2$ Li(OH)	1.13 μg_{Pt} cm^{-2}	N/A	2.28	N/A	N/A	106	10
Pt(111)/$Co(OH)_2$	N/A	N/A	~0.5	N/A	N/A	159	11
Ni_5P_4	50000	1.2 (η=13-21 mV) 4.2 (η=21-76 mV) normalized by BET area	N/A	13	N/A	~63 (normalized by BET area)	12
NiMo nanopowers	1000	N/A	N/A	~7	N/A	N/A	13
***hcp*-excavated Pt-Ni nano-multipods**	**7.65 μg_{Pt} cm^{-2}**	**1.65**	**11.1**	**22.5**	**3.03**	**38**	**This work**

m: (μg cm^{-2}): mass of the catalyst loading on the electrode.

j_o (mA cm^{-2}): exchange current density normalized to the ECSA.

j_s (mA cm^{-2}): current density normalized to the ECSA at η=70 mV.

j_g (mA cm^{-2}): current density normalized to the geometric area at η=70 mV.

j_m (mA $\cdot\mu g_{Pt}^{-1}$): current normalized to the mass of Pt on the electrode at η=70 mV.

η (mV): overpotential at j_s=4 mA cm^{-2}.

Supplementary Note 1. *Rietveld* refinement.

For the *Rietveld* refinement, the XRD pattern was collected at a scan rate of 2 degree/min in order to get high quality data. The Rietveld refinement was conducted by using Topas software. Due to the fact that a small diffraction peak appeared at 48.6 °(corresponding to the *fcc* phase), we applied multi-phase simulation. The structure models of *fcc* and *hcp* phases corresponded to $Pt_{56}Ni_{44}$ and $Pt_{12}Ni_{88}$, respectively. In the refinement, the cell parameters and phase contents were refined, while the atomic occupancy factors were not refined. The refinement results demonstrated that the final product was composed of 99.8 wt% *hcp* phase and 0.2 wt% *fcc* phase. The small amount of *fcc* phase could be *fcc* crystal seeds formed at the early stage. As the content of *fcc* PtNi alloy phase is very low in the products, it would not affect the properties of *hcp* Pt-Ni multipods.

Supplementary Note 2. Calculation about adsorption energies of oleic acid.

A (4×3) five-layer model and a (4×2) five-layer model are conducted to simulate the {0001} and $\{11\bar{2}0\}$ facets, respectively. The surface areas per unit cell of these two models are similar, 71.4 $Å^2$ for {0001} surface and 78.4 $Å^2$ for $\{11\bar{2}0\}$ surface. We assume that each unit cell could contain two oleic acid (OAH) molecules, resulting in similar packing density. The adsorption energy of OAH can be calculated as:

$$\Delta E_{OAH} = \{(E_{DFT}(Slab/OAH)\text{-}E_{DFT}(Slab) - 2\times E_{DFT}(OAH)\}/2 \quad (1)$$

in which E_{DFT}(Slab/OAH) is the energy of the slab with two OAH molecules; E_{DFT}(Slab) is the energy of the slab (*hcp*-Ni{0001} or $\{11\bar{2}0\}$), and E_{DFT} (OAH) is the energy of gas phase OAH. Here, we neglect *van der Waals* interactions between the alkyl chains and the entropic contribution in the solvent, because these effects could approximately cancel each other when comparing different surfaces. We consider not only the molecular adsorption (OAH) but also the dissociative adsorption (OA+H), as shown in Supplementary Table 2, and corresponding optimized structures are illustrated in Supplementary Figure 24.

Geometrically, Ni atoms on the $\{11\bar{2}0\}$ are seven-coordinated, less saturated than {0001}. More open surface usually results in stronger adsorption. Actually, our DFT calculations demonstrate the adsorptions on the $\{11\bar{2}0\}$ surface are favored over those on the {0001} by ~0.25 eV, either the molecular adsorption or dissociated adsorption. This finding indicates that $\{11\bar{2}0\}$ surface could be stabilized by the adsorption of OAH, being consistent with the experimental observations.

Supplementary Note 3. Calculation about deposited energies of Pt or Ni atoms.

Computationally, a (3×3) surface with five layer slabs is built, and the top three layers are allowed to be relaxed. For simplicity, we use experimental cell parameters obtained from bulky *fcc* Pt-Ni alloy, and assume the utmost layer of Pt-Ni {111} is completely occupied by Pt atoms, i.e. Pt skin. Under the Pt skin, Pt atoms are alloying with Ni atoms in the manner of half to half.

Here, two possible models are considered, c.f. Supplementary Figure 25. In Model I, we assume that Ni (or Pt) atom would be free of solvation, and surface would be clean (free of modifier). In this case, the deposition energy can be expressed as:

$$\Delta E_d = \{E_{DFT}(Slab/n\times M)\text{-}E_{DFT}(Slab) - n\times E_{DFT}(M)\}/n \qquad (2)$$

in which E_{DFT}(Slab/n×M) is denoted as the energy of the slab with n metal atoms; E_{DFT}(Slab) the energy of the slab; E_{DFT} (M) the energy of isolate metal atom and n is the number of metal atoms. For single atom deposition (1/9 ML), the ΔE_d(Ni) is predicted to be exothermic by 3.65 eV, while the ΔE_d(Pt) 3.82 eV. When the coverage of Ni atom increases to 1.0 ML, the ΔE_d(Ni) is enlarged to be -4.49 eV/atom, which could be attributed to the formation of Ni-Ni bonds. Similarly, increasing the Pt coverage also enhances the ΔE_d(Pt) (-5.35 eV/atom at 1.0 ML). Thus, preferential deposition of Pt atoms over Ni atoms takes place, in contradictory with experimental results.

Model II describes a deposition of organic N coordinated metal atom on the organic N coated Pt skin surface. Experimentally, we use oleylamine as solvent. For the sake of computational efficiency, octylamine is adopted as a reduced model for oleylamine. DFT calculations show that both Ni and Pt atom can be coordinated with two amine (denoted as $M(Am)_2$) molecules, with the binding energy of -3.08 eV and -4.08 eV, respectively. In previously works, we estimate the coverage of oleylamine on the surface of Pt-Co alloy is around 0.12 ML-0.15 ML because oleylamine is bulky and adsorbed on the surface with a tilt mode.[14] Here, we assume the coverage of amine would attain 1/9 ML. In this case, the binding energy of amine is predicted to be -0.74 eV. From above calculations, we infer that not only the Ni (or Pt) atom but the alloy surface could not be 'clean' under experimental condition. In Model II, with $M(Am)_2$ approaching, M-Pt bonds are forming while two amine molecules are leaving from surface, to keep the 1/9 ML coverage of amine. Similar to Eq(2), the deposition energy in Model II can be written as:

$$\Delta E_d = \{E_{DFT}(Slab\text{-}Am/n\times M)+2n\times E_{DFT}(Am)\text{-}E_{DFT}(Slab\text{-}Am) - n\times E_{DFT}(M(Am)_2)\}/n \qquad (3)$$

in which E_{DFT}(Slab-Am /n×M) is represented as the energy of the slab with one amine and n M atoms; E_{DFT}(Am) the energy of amine; E_{DFT}(Slab-Am) the energy of the slab with one amine; and E_{DFT} ($M(Am)_2$) the energy of isolate $M(Am)_2$ complex. To our surprise, according to Eq(3), ΔE_d(Ni) become more favorable than ΔE_d(Pt), either for single atom deposition (-1.40 eV *v.s.* -0.59 eV) or monolayer deposition (-1.45 eV *v.s.* -1.30 eV). The reverse preference of deposition might be due to that the Pt atoms would have a much lower chemical potential than Ni atoms. We expect that Pt atoms could be precipitated in a solvent with weak coordination ability. Indeed, it is observed experimentally that when octadecene is used as solvent, metallic Pt can be detected by EDS, echoing with our theoretical view.

Supplementary References

(1) Subbaraman, R.; Tripkovic, D.; Strmcnik, D.; Chang, K.-C.; Uchimura, M.; Paulikas, A. P.; Stamenkovic, V.; Markovic, N. M. Enhancing hydrogen evolution activity in water splitting by tailoring Li^+-Ni $(OH)_2$-Pt interfaces. *Science* **334**, 1256 (2011).

(2) Rheinländer, P.; Henning, S.; Herranz, J.; Gasteiger, H. A. Comparing hydrogen oxidation and evolution reaction kinetics on polycrystalline platinum in 0.1 M and 1 M KOH. *ECS Trans.* **50**, 2163 (2013).

(3) Danilovic, N.; Subbaraman, R.; Strmcnik, D.; Chang, K.-C.; Paulikas, A. P.; Stamenkovic, V. R.; Markovic, N. M. Enhancing the Alkaline Hydrogen Evolution Reaction Activity through the Bifunctionality of $Ni(OH)_2$/Metal Catalysts. *Angew. Chem. Int. Ed.* **51**, 12495 (2012).

(4) Sheng, W.; Myint, M.; Chen, J. G.; Yan, Y. *Energy Environ. Sci.* **6**, 1509 (2013).

(5) Chen, C.; Kang, Y.; Huo, Z.; Zhu, Z.; Huang, W.; Xin, H. L.; Snyder, J. D.; Li, D.; Herron, J. A.; Mavrikakis, M.; Chi, M.; More, K. L.; Li, Y.; Markovic, N. M.; Somorjai, G. A.; Yang, P.; Stamenkovic, V. R. Highly crystalline multimetallic nanoframes with three-dimensional electrocatalytic surfaces. *Science* **343**, 1339 (2014).

(6) Zheng, Y.; Jiao, Y.; Li, L. H.; Xing, T.; Chen, Y.; Jaroniec, M.; Qiao, S. Z. Toward design of synergistically active carbon-based catalysts for electrocatalytic hydrogen evolution. *ACS Nano*. **8**, 5290 (2014).

(7) Zheng, Y. *et al.* Hydrogen evolution by a metal-free electrocatalyst. *Nat. Commun.* **5**, 3783 (2014).

(8) Yin, H. *et al.* Ultrathin platinum nanowires grown on single-layered nickel hydroxide with high hydrogen evolution activity. *Nat. Commun*. **6**, 6430 (2015).

(9) Tang, M. H.; Hahn, C.; Klobuchar, A. J.; Ng, J. W. D.; Wellendorff, J.; Bligaard, T.; Jaramillo, T. F. Nickel–silver alloy electrocatalysts for hydrogen evolution and oxidation in an alkaline electrolyte. *Phys. Chem. Chem. Phys.* **16**, 19250 (2014).

(10) Wang, L.; Lin, C.; Huang, D.; Chen, J.; Jiang, L.; Wang, M.; Chi, L.; Shi, L.; Jin, J. Optimizing the Volmer Step by Single-Layer Nickel Hydroxide Nanosheets in Hydrogen Evolution Reaction of Platinum. *ACS Catal.* **5**, 3801 (2015).

(11) Subbaraman, R. *et al.* Trends in activity for the water electrolyser reactions on 3d M(Ni,Co,Fe,Mn) hydr(oxy)oxide catalysts. *Nat. Mater*. **11**, 550 (2012).

(12) Laursen, A. B. *et al.* Nanocrystalline Ni_5P_4: A hydrogen evolution electrocatalyst of exceptional efficiency in both alkaline and acidic media. *Energy Environ. Sci.* **8**, 1027 (2015).

(13) McKone, J. R. *et al.* Ni−Mo Nanopowders for Efficient Electrochemical Hydrogen Evolution. *ACS Catal.* **3**, 166 (2013)

(14) Wu, B. Zheng, N. Fu, G. Selective hydrogenation of *alpha, beta*-unsaturated aldehydes catalyzed by amine-capped platinum-cobalt nanocrystals. *Angew. Chem. Int. Ed*. **51**, 3440 **(**2012).
